# The Role of Photolabile Dermal Nitric Oxide Derivates in Ultraviolet Radiation (UVR)-Induced Cell Death

**DOI:** 10.3390/ijms14010191

**Published:** 2012-12-21

**Authors:** Christian Opländer, Christoph V. Suschek

**Affiliations:** 1 Department of Plastic and Reconstructive Surgery, Hand Surgery, and Burn Center, Medical Faculty, RWTH Aachen University, Pauwelsstr. 30, D-52074 Aachen, Germany; 2 Department of Trauma and Hand Surgery, Medical Faculty of the Heinrich-Heine-University, 40225 Düsseldorf, Germany; E-Mail: suschek@uni-duesseldorf.de

**Keywords:** UVA, nitrite, nitric oxide, cell death, lipid peroxidation, apoptosis, necrosis, antioxidants

## Abstract

Human skin is exposed to solar ultraviolet radiation comprising UVB (280–315 nm) and UVA (315–400 nm) on a daily basis. Within the last two decades, the molecular and cellular response to UVA/UVB and the possible effects on human health have been investigated extensively. It is generally accepted that the mutagenic and carcinogenic properties of UVB is due to the direct interaction with DNA. On the other hand, by interaction with non-DNA chromophores as endogenous photosensitizers, UVA induces formation of reactive oxygen species (ROS), which play a pivotal role as mediators of UVA-induced injuries in human skin. This review gives a short overview about relevant findings concerning the molecular mechanisms underlying UVA/UVB-induced cell death. Furthermore, we will highlight the potential role of cutaneous antioxidants and photolabile nitric oxide derivates (NODs) in skin physiology. UVA-induced decomposition of the NODs, like nitrite, leads not only to non-enzymatic formation of nitric oxide (NO), but also to toxic reactive nitrogen species (RNS), like peroxynitrite. Whereas under antioxidative conditions the generation of protective amounts of NO is favored, under oxidative conditions, less injurious reactive nitrogen species are generated, which may enhance UVA-induced cell death.

## 1. Introduction

**Figure 1:**
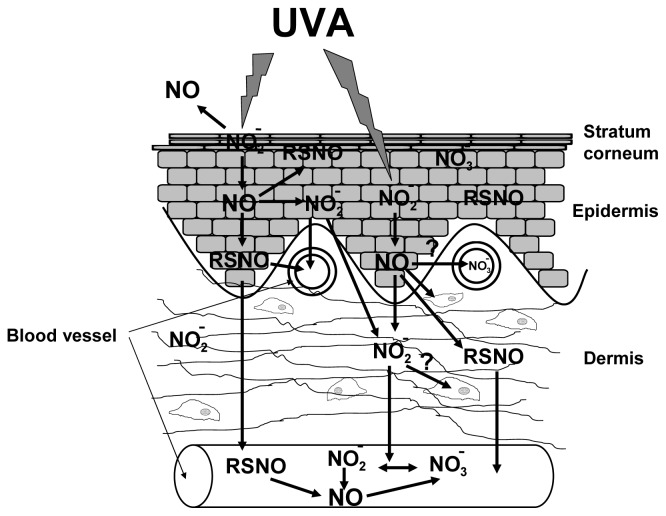
UVA-induced mobilization of dermal nitric oxide derivates. UVA-induced nitrite (NO_2_^−^) decomposition in the skin leads to the generation of nitric oxide (NO) and nitroso compounds (RSNO). NO can diffuse into blood vessels, where it is oxidized to nitrate by hemoglobin, or into deeper skin levels, where it is oxidized to nitrite. RSNO are vasoactive and can enter the blood system, evolving changes in blood flow and pressure.

Human skin is exposed to sunlight on a daily basis, and especially, the biological effects of the ultraviolet region namely UVB (280–315 nm) and UVA (315–400 nm) have been investigated intensely in the last decades. It has been recognized that exposure to solar ultraviolet radiation has both beneficial and deleterious effects on human health and skin physiology. Apart from painful sunburns as a result of excessive sun exposure, chronic exposure to UVA and UVB is related to increased risk of skin cancer [1] and premature skin ageing [2]. On the other hand, a growing body of studies and data suggest general health benefits of sunlight and UVR-exposure [3]. In this context, in particular, the production of Vitamin D in the deeper epidermis of the skin by UVB and the resulting higher circulating concentrations of serum 25-hydroxyvitamin D may reduce the risk of many chronic and infectious diseases, *inter alia*, cancer, hypertension and cardiovascular diseases, autoimmune diseases and bacterial and viral infections [4–6]. Furthermore, it has been suggested that many of the beneficial effects of sunlight, particularly those related to cardiovascular health, are mediated by mechanisms that are independent of vitamin D production. For example, it has been hypothesized that photolabile nitric oxide (NO)-related species or compounds, such as nitrite and nitrosothiols, which are stored in comparably high concentration in the skin, can be mobilized by UVA and delivered to the systemic circulation, exerting coronary vasodilation and antihypertensive effects (see Figure 1) [7,8].

However, despite the controversial discussion of the beneficial and hazardous effects of sunlight, there is incontrovertible evidence that UVB, and also UVA exposure, can induce cell damage, which in turn causes necrosis or induces apoptosis [9] and autophagy [10,11]. Thus, we will review briefly in this article the common mechanism of UVA- and UVB-induced cell damage and highlight the possible role of NO-related species as endogenous photo-sensitizers, which, especially during UVA exposure, may represent a protective principle against UVA-induced and ROS-mediated cell and tissue damage.

## 2. UVR-Induced Cell Damage

An overexposure to UVB can lead to all kinds of maladies, including sunburn, skin cancer and cataracts. It is generally accepted that the mutagenic and carcinogenic properties of UVB is due to the direct interaction with DNA and consequent generation of pyrimidine dimers, namely cyclobutane pyrimidine dimers (CPDs) and pyrimidine pyrimidone photoproducts [12].

Studies suggest that these lesions are involved in photocarcinogenesis by the high proportion of p53 mutations (TC to TT or CC to TT transitions) detected at bipyrimidine sites in non-melanoma skin cancers [13] and UVB-induced skin tumors in a hairless mouse model [14].

Although these DNA-damages are potentially mutagenic, virtually all mammalian cells have several enzymatic systems to repair UVB-induced DNA [15]. Once cell cycle checkpoint systems sense abnormal chromosomal DNA structures, they execute cell cycle arrest through inhibiting the activity of cell cycle regulators. This is necessary to coordinate cell division with the DNA repair process or, if the damage is too severe, execute apoptosis and premature senescence of the affected cells [16].

However, UVB-stress response, such as NF-kappa B activation, occurs also in enucleated cells, indicating that the response is initiated at or near the plasma membrane, rather than the nucleus [17]. Further studies could support these findings and show that a major part of the UVB-response of cells is DNA-damage independent. As very early initiating events after UVB-exposure, the clustering and internalization of cell surface receptors for epidermal growth factor (EGF), tumor necrosis factor (TNF) and interleukin-1β (IL-1β) could be observed [18]. As one of the responsible UVB chromophores for DNA-independent UVB-response, the arylhydrocarbon receptor (AhR) ligand 6-formylindolo[3,2-*b*]carbazole (FICZ) was identified in human skin cells [19]. Recent studies have shown that UVB irradiation also induces in keratinocytes the expression of Cyclophilin D, a key component for opening mitochondrial permeability transition pores, leading to both apoptopic and necrotic cell death [20]. As reported recently, UVB irradiation modificates major lipid raft components in skin cells by increasing cholesterol levels in membrane rafts, which leads to Fas-receptor aggregation and to subsequent events as the formation of death-inducing signaling complex and apoptosis [21]. On the other hand, it has been demonstrated that UVB irradiation leads also to an increase of the protein prohibitin in membrane rafts, which may have a protective function against UVB-induced apoptosis [22]. Apart from the UVA-induced effects on DNA and cell membranes, UVB affects miRNA expression profiles in mouse epidermis, which could be associated with photocarcinogenesis and apoptosis [23]. Moreover, UVB as well as UVA can induce damage of proteins mainly by interaction with amino acid residues, such as tryptophan, tyrosine, histidine and cystine, via both excited state species and radicals [24].

To sum up, UVB response in the skin is a multifaceted biological process and originates from multiple intracellular sites. Besides the above mentioned mechanism, UVB can induce also the formation of reactive oxygen species [25] and affects or damages proteins by direct oxidation or by covalent binding of lipid peroxidation breakdown products [26,27].

The amount of UVA radiation on earth’s surface is approximately 20-fold higher than that of UVB. It penetrates deeper (up to 1 mm) into the skin in comparison to UVB (20 μM) and can reach the dermis [28]. UVA is responsible for the tanning effects of human skin and had been considered mostly harmless for many years. However, studies have shown that UVA contributes to photoaging, photocarcinogenesis and photodermatosis [29,30] by photooxidative mechanisms and the formation of reactive oxygen species (ROS) [31]. Furthermore, apart from UVB radiation, UVA radiation can also induce the generation of bipyrimidine photoproducts, respectively, cyclobutane pyrimidine dimers (CPDs). It seems now most likely that UVA-induced CPDs arise from a direct photoreaction rather than photosensitizer-mediated processes [32]. The formation of ROS, for example, oxygen radicals and singlet oxygen seems to be dependent on non-DNA chromophores, such as porphyrins, bilirubin, melanin and flavins, acting as endogenous photosensitizers. Excessive ROS formation in a cell leads to the damage of many biomolecules, including DNA and membrane lipids and, thus, cytotoxicity, mutations and alterations in cell signaling pathways [33]. Given solar spectral distribution, solar UVA radiation is much more effective in triggering lipid peroxidation than UVB [34]. UVA-induced oxidative stress provokes an immediate increase in the available pool of intracellular redox-active and chelatable iron ions, leading to the catalytic formation of more oxygen-derived free radicals that may overwhelm the cellular antioxidant defense, with subsequent cell damage [35].

Regarding UVA-induced cell membrane damage, it had been shown that the radical nitric oxide can inhibit lipid peroxidation by scavenging lipid peroxyl radicals and/or inhibiting initiators of lipid peroxidation, respectively, and, consequently, also inhibits UVA-induced cell death via apoptosis or necrosis [36–40]. However, NO may react with superoxide to peroxynitrite, which initiates lipid peroxidation itself and oxidizes lipid soluble antioxidants [41].

Therefore, the next sections will focus on the involvement of NO and photolabile NO-related species in the physiological response of the skin to UVA-exposure.

## 3. Physiology of Nitric Oxide

Nitric oxide (NO) plays a pivotal role in human’s physiology and pathophysiology [42–44]. It is the smallest known bioactive product of mammalian cells, is highly diffusible and reactive and can be produced by most cell types. Although ubiquitous, this simple molecule can act very specifically, controlling vital functions, such as neurotransmission and vascular tone via activation of soluble guanylyl cyclase [45,46], gene transcription [47] and mRNA translation (via iron-responsive elements) [48]. It can generate posttranslational modifications of proteins (via ADP-ribosylation) [49] and is capable of destroying tumor cells and parasites by inhibiting iron-containing enzymes [50] or interacting directly with the DNA of these cells [51]. Nitric oxide plays an important role in skin physiology as well and is involved in wound healing [52], keratinocyte proliferation and differentiation [53] and cutaneous inflammation and immune reactions [54]. In addition, several lines of evidence indicate that NO regulates the expression of protective stress response genes, such as vascular endothelial growth factor (VEGF) and heme oxygenase (HO)-1 or Bcl-2 [38,55,56]. Furthermore, NO can inhibit UVA-induced lipid peroxidation [41,57], and the coordinated effects of NO on gene expression and preservation of membrane integrity effectively protect against UVA- and reactive oxygen species (ROS)-induced apoptotic as well as necrotic cell death [39].

In the human body, NO is formed endogenously by three NO synthase enzymes [58]. The keratinocytes express the neuronal isoform of NO synthase (nNOS), whereas the fibroblasts and other cell types in the skin express the endothelial isoform (eNOS). This constitutive low level NO production in the skin seems to play a role in the maintenance of barrier function and in determining blood flow [59]. The inducible isoform of NO synthase (iNOS) is not expressed usually in the skin, but under certain conditions, virtually all skin cells are capable of expressing iNOS. The following high level production of NO is often correlated with psoriasis and other inflammatory skin conditions [60]. The irradiation of the skin by UVB and/or UVA induces in the skin the release of inflammation transmitters, like IL-1, IL-10, TGF-β1, TNF-α, which induce iNOS to produce higher NO-concentration, leading to erythema, edema and stimulation of melanogenesis (for review see [61]). However, we could show that iNOS can be induced by UVA in the absence of proinflamatory cytokines [62]. Hereinafter, we will explain the many functions of NO and highlight the potential role of naturally occurring NODs in the biological response to UVA challenge by non-enzymatic formation of NO.

## 4. Nitrogen Oxide Derivates as Source of Nitric Oxide

A part of the NO molecules generated in skin reacts rapidly with oxygen or proteins. The products of these reactions are mainly *S*- or *N*-nitroso compounds (RSNOs or RNNOs) and the oxidation products nitrite or nitrate. These nitrogen oxide derivates (NODs) stay close to the place of their origin and accumulate this way in cells and tissue. Thus, comparably high NOD concentrations can be found in human skin (nitrite up to 15 μM, nitroso compounds up to 7 μM and nitrate up to 100 μM) [63].

Additionally to the enzymatic NO formation as described above, NO can also be formed non-enzymatically, e.g., by pH-catalyzed and UVA-induced decomposition of NODs, namely nitrite, nitrate and *S*-nitroso compounds (RSNOs), such as *S*-nitrosoalbumin, *S*-nitrosoglutathione or *S*-nitrosocysteine.

Already in the 1960s, the pioneers of nitric oxide research, Furchgott and Ehrreich, accidentally made the observation that daylight irradiation of blood vessels induces dilation [64]. This effect called photorelaxtion was markedly enhanced by nitrite solutions, indicating that under certain circumstances, nitrite may exhibit relaxation activities comparable to NO.

**Figure 2:**
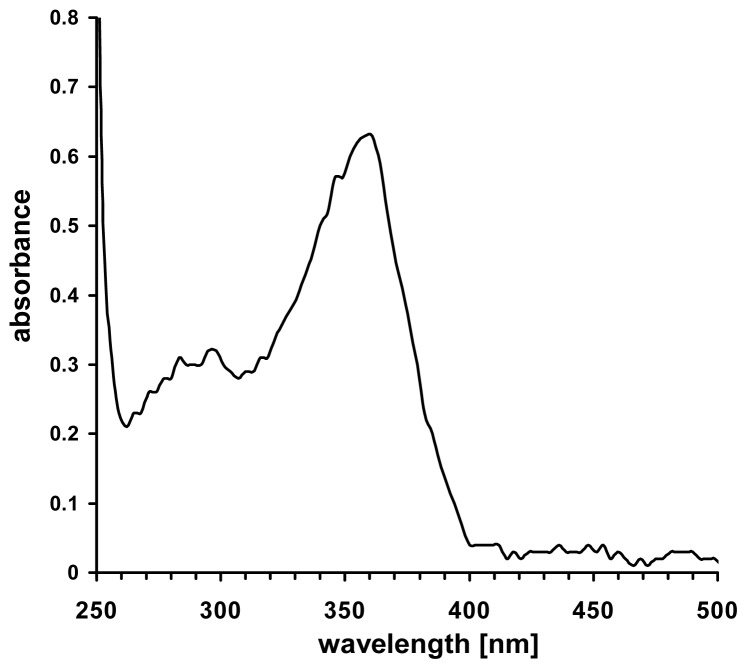
UV/VIS-spectrum of an aqueous sodium nitrite solution (25 mM).

Indeed, studies in environmental chemistry revealed that the N-O-bond of the nitrite ion in aqueous solution will be disrupted by the energy of light within the UVA spectrum at 340–360 nm (see Figure 2).

This UVA-induced photodecomposition of nitrite results in a constant low-level formation of NO and also of various reactive oxygen and nitrogen species [65]. In comparison, the irradiation of RSNOs results in a higher, but short-lasting, NO-formation, accompanied by the rapid loss of these nitroso species [66]. Nitrate is generally considered as a very inert oxidative product of NO. But it could be shown that limited amounts of NO are produced during UV-irradiation of nitrate, which is enhanced in the presence of thiols [67].

However, we consider nitrite as the predominant source of NO during UVA exposure of the human skin. In the next sections, we will describe the underlying mechanism of UVA-induced nitrite decomposition, its rule in cell death and protection and how antioxidants affect the yield of bioactive NO.

## 5. UVA Induces Non-Enzymatic NO-Formation in Human Skin

Nitric oxide derivates are present in blood as products of enzymatic NO synthesis and as a consequence of the intake of nitrite/nitrate-containing food. In normal human skin, we found nitrite and *S*-nitrosothiols (RSNO) at concentrations 25- or 360-fold higher than those found in the plasma of healthy volunteers [63]. This finding could be confirmed by Mowbray *et al*. [68] using other methods for the determination of cutaneous NODs.

The challenge for normal human skin samples with UVA at doses equivalent to only 3–5 min of sun exposure in the Central European summer (*approx*. 5 mW/cm^2^) leads to significant non-enzymatic NO-formation due to the decomposition of nitrite and RSNOs [63]. This result could be found also *in vivo* by Mowbray *et al*. [68] and by ourselves in further studies, where we measured *in vivo* the enzyme-independent generation of gaseous NO directly above the skin surface [40]. Surprisingly, nitrate does not contribute to UVA-provoked NO release from human skin.

Previously, we had shown that endogenously produced iNOS-derived NO and exogenously applied NO shows protective activity against UVA-induced cell death in endothelial cells [38,39]. Further studies showed that also the presence of nitrite exerts NO-dependent protection against UVA-induced cell death in endothelial cells [37].

The skin is frequently exposed to UVA irradiation and UVA especially reaches the dermis and causes photo-oxidative stress in the resident fibroblasts. The purpose of further studies was to examine whether nitrite or other NODs may exhibit a similar protective NO-dependent activity against UVA-induced cell death in fibroblasts as in endothelial cells. In contrast to endothelial cells, we found that the presence of exogenous nitrite enhances the UVA-induced lipid peroxidation and the susceptibility to toxic effects of UVA in dermal fibroblast in a concentration-dependent manner [69].

The mechanism of UVA-induced nitrite decomposition (Reactions 1–5) reveals the initial generation of NO and a cascade of further reactions leading to nitrosative stress by generation of the highly toxic nitrogen dioxide NO_2_·, which is capable of initiating the lipid peroxidation chain reaction.

(1) $${{\text{NO}}_{2}}^{-}+{\text{hv}}_{365\text{nm}}\to \text{NO}\cdot +\text{O}\cdot^{-}$$

(2) $$\text{O}\cdot^{-}+{\text{H}}_{2}\text{O}\to \text{OH}\cdot +{\text{OH}}^{-}$$

(3) $${{\text{NO}}_{2}}^{-}+\text{OH}\cdot \to {\text{NO}}_{2}\cdot +{\text{OH}}^{-}$$

(4) $${\text{NO}}_{2}\cdot +\text{NO}\cdot ↔{\text{N}}_{2}{\text{O}}_{3}$$

(5) $${\text{N}}_{2}{\text{O}}_{3}+{\text{H}}_{2}\text{O}\to 2\;{{\text{NO}}_{2}}^{-}+2\;{\text{H}}^{+}$$

**Figure 3:**
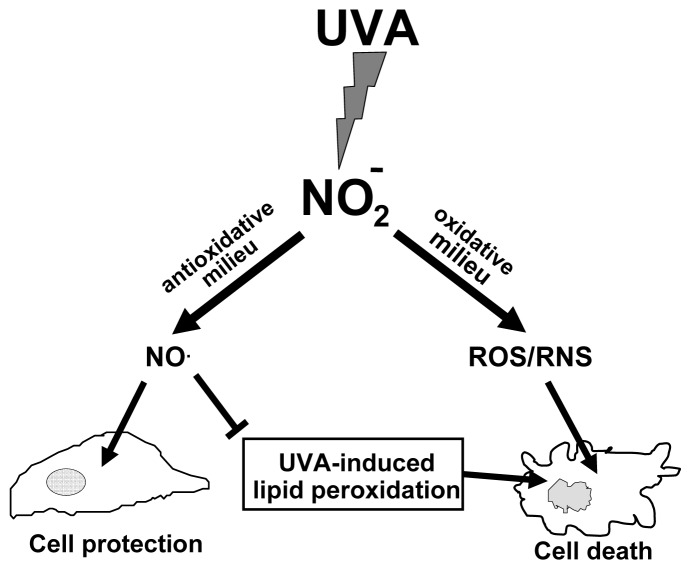
The UVA-induced decomposition of nitrite (NO_2_^−^) can have different effects on lipid peroxidation and cell viability dependent on the redox state of the cell milieu. During UVA-exposure, an antioxidative milieu leads to a higher yield of protective NO by UVA-induced nitrite decomposition, whereas oxidative stress and low concentrations of antioxidants decrease the NO-yield and cause cell damage by the prevailing generation of reactive nitrogen species (RNS).

Furthermore, nitrogen dioxide consumes NO (Reaction 4), which is an efficient inhibitor of lipid peroxidation. Thus, Reaction (4) further supports the injurious effects of UVA-induced nitrite decomposition. Consequently, ascorbic acid, a potent nitrogen dioxide antagonist [70], protects against UVA/nitrite-induced lipid peroxidation and toxicity by scavenging nitrogen dioxide and by the simultaneous enhancement of UVA-induced NO formation from nitrite. Thus, the oxidative milieu is crucial for the outcome of UVA-induced nitrite decomposition and its effects on cell viability [69]. An overview is given in Figure 3.

**Figure 4:**
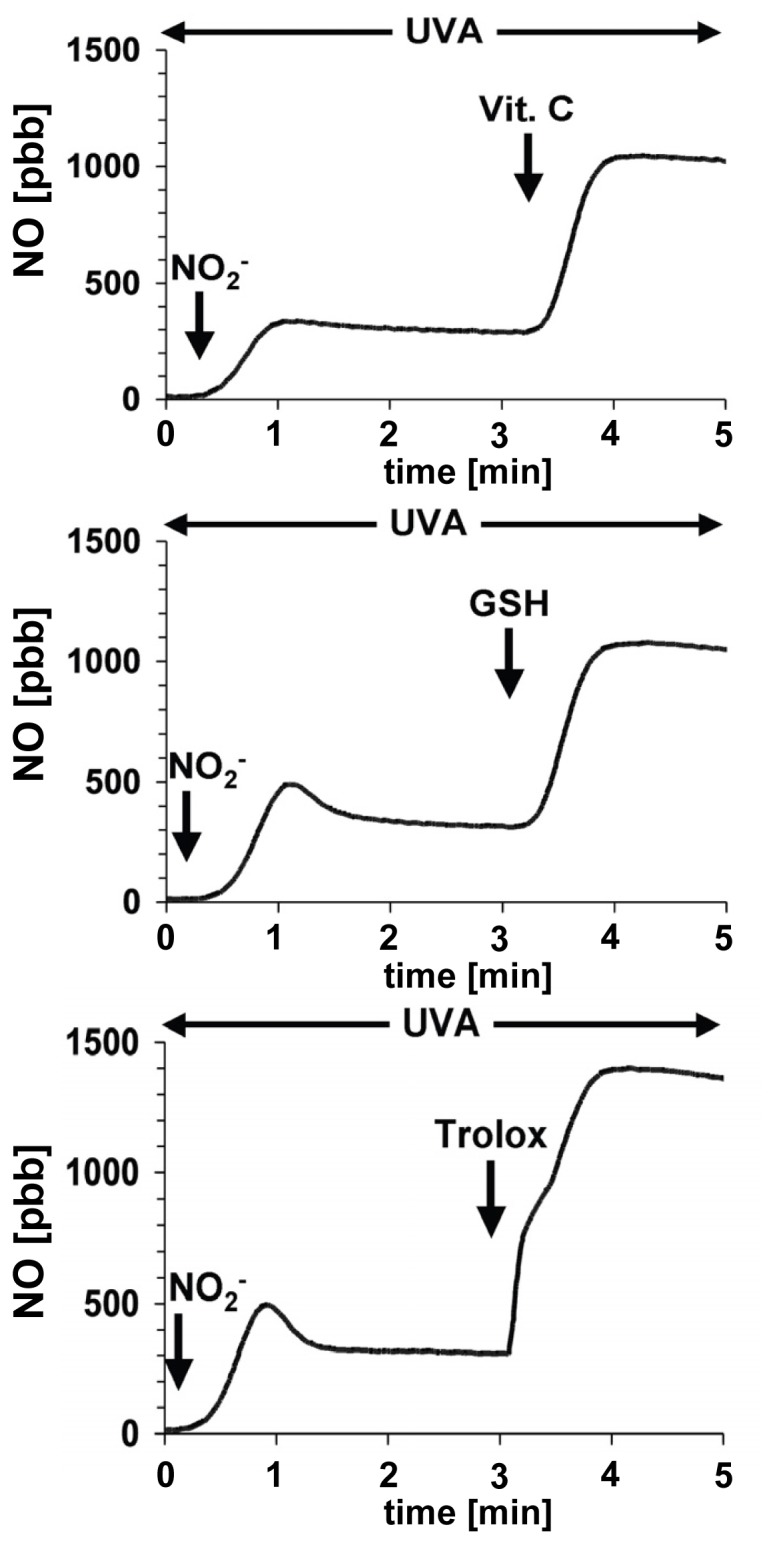
Physiological concentrations (100 μM) of antioxidants, such as vitamin C, glutathione (GSH) or trolox, can enhance significantly the NO-yield obtained from UVA-induced nitrite decomposition (10 μM).

However, other antioxidants, like glutathione and trolox, a water-soluble vitamin E derivate, are also capable of enhancing the NO-formation from UVA-induced nitrite decomposition by many fold (see Figure 4).

But, in contrast to ascorbic acid, the presence of glutathione or trolox leads to an increase of UVA/nitrite-induced necrotic cell death. The reaction of glutathione with nitrogen dioxide preferably produces sulfur-centered glutathionyl radicals, which have been shown to promote the oxidation of phospholipids [71]. The initial product of the reaction of nitrogen dioxide with α-tocopherol, the α-tocopheroxyl radical, is an effective lipid peroxidation-inducing agent in living cells and causes cell death [72,73].

On the other hand, the addition of NO can reverse these toxic effects. In conclusion, oxidative and nitrosative stress can result from an imbalance of prooxidants and antioxidants with excessive, destructive free-radical chemistry. Here, it seems that endothelial cells have a higher antioxidative capacity against reactive nitrogen species than fibroblasts. With respect to the possible high concentration of nitrite in sweat and the skin surface, the use of vitamin E containing creams before UVA exposure may be a potential hazard to the skin.

## 6. The Protective Role of Intracellular Nitrite during UVA-Challenge

Although the presence of supraphysiological concentrations of nitrite during UVA exposure shows enhanced toxicity in the cell culture of human dermal fibroblasts, nothing is known about the impact of intracellular naturally occurring NODs.

With respect to the important role of NO in human cutaneous physiology and the possible protective activity of NO, we examined the influence of intracellularly-present photolabile NODs on the UVA-induced toxicity of human skin fibroblasts [74].

We could show an intracellular NO-formation during UVA-irradiation, which is dependent on the intracellular NOD-concentration. We found that intracellular photolabile NODs, generally nitrite, modulate UVA-induced cell damage. A depletion of intracellular NODs leads to a decrease of intracellular NO-formation, accompanied by an increase of lipid peroxidation and UVA-induced cell toxicity via necrotic cell death. Supplementation with nitrite in physiological concentrations can reverse completely the depletion and the increased susceptibility of NOD-depleted fibroblasts cultures to the injurious effects of UVA. The use of cPTIO as NO-scavenger proves the protective role of NO produced non-enzymatically by nitrite decomposition.

**Figure 5:**
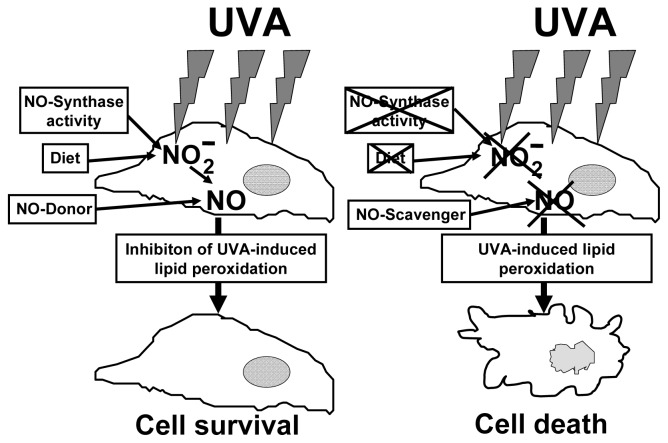
Intracellular nitrite in physiological concentrations has protective effects against UVA. Depletion of nitrite by inhibiting nitric oxide synthase activity and nitrite free supplementation or scavenging NO increases lipid peroxidation and cell death significantly.

These findings indicate that non-enzymatic NO formation due to decomposition of intracellularly occurring photolabile NODs represents an elementary and fast reacting physiological principle of protection against UVA-induced lipid peroxidation and cell death (see Figure 5). Additionally, studies have shown that NO may inhibit autophagy of cells [75]. Since UVB irradiation can induce autophagy in epidermal cells, theoretically, the UVA-induced generation of NO might protect also against a possible UVB-induced autophagy in skin cells [10] during exposure to sun light.

In conclusion, targeting dermal NODs by pharmacological or dietary interventions may represent an additional approach in preventing UVA-, and maybe also UVB-, induced skin damage. However, further studies are necessary for the investigation of the role of nitrite and other NODs in skin physiology, their potential use as agents for sun screens and if a nitrite/nitrate enriched diet can influence the dermal NOD-stores and the UVA/UVB-response.
